# Exome Sequencing Identifies Rare Deleterious Mutations in DNA Repair Genes *FANCC* and *BLM* as Potential Breast Cancer Susceptibility Alleles

**DOI:** 10.1371/journal.pgen.1002894

**Published:** 2012-09-27

**Authors:** Ella R. Thompson, Maria A. Doyle, Georgina L. Ryland, Simone M. Rowley, David Y. H. Choong, Richard W. Tothill, Heather Thorne, kConFab, Daniel R. Barnes, Jason Li, Jason Ellul, Gayle K. Philip, Yoland C. Antill, Paul A. James, Alison H. Trainer, Gillian Mitchell, Ian G. Campbell

**Affiliations:** 1 Victorian Breast Cancer Research Consortium Cancer Genetics Laboratory, Peter MacCallum Cancer Centre, East Melbourne, Victoria, Australia; 2 Bioinformatics Core Facility, Peter MacCallum Cancer Centre, East Melbourne, Victoria, Australia; 3 Centre for Cancer Research, Monash Institute of Medical Research, Monash University, Clayton, Victoria, Australia; 4 Molecular Genomics Core Facility, Peter MacCallum Cancer Centre, East Melbourne, Victoria, Australia; 5 Kathleen Cunningham Foundation Consortium for Research into Familial Breast Cancer (kConFab), Peter MacCallum Cancer Centre, East Melbourne, Victoria, Australia; 6 Centre for Cancer Genetic Epidemiology, Department of Public Health and Primary Care, University of Cambridge, Cambridge, United Kingdom; 7 Life Sciences Computation Centre, Victorian Life Sciences Computation Initiative, Carlton, Victoria, Australia; 8 Familial Cancer Centre, Peter MacCallum Cancer Centre, East Melbourne, Victoria, Australia; 9 Sir Peter MacCallum Department of Oncology, University of Melbourne, Parkville, Victoria, Australia; 10 Department of Pathology, University of Melbourne, Parkville, Victoria, Australia; University of Washington, United States of America

## Abstract

Despite intensive efforts using linkage and candidate gene approaches, the genetic etiology for the majority of families with a multi-generational breast cancer predisposition is unknown. In this study, we used whole-exome sequencing of thirty-three individuals from 15 breast cancer families to identify potential predisposing genes. Our analysis identified families with heterozygous, deleterious mutations in the DNA repair genes *FANCC* and *BLM*, which are responsible for the autosomal recessive disorders Fanconi Anemia and Bloom syndrome. In total, screening of all exons in these genes in 438 breast cancer families identified three with truncating mutations in *FANCC* and two with truncating mutations in *BLM*. Additional screening of *FANCC* mutation hotspot exons identified one pathogenic mutation among an additional 957 breast cancer families. Importantly, none of the deleterious mutations were identified among 464 healthy controls and are not reported in the 1,000 Genomes data. Given the rarity of Fanconi Anemia and Bloom syndrome disorders among Caucasian populations, the finding of multiple deleterious mutations in these critical DNA repair genes among high-risk breast cancer families is intriguing and suggestive of a predisposing role. Our data demonstrate the utility of intra-family exome-sequencing approaches to uncover cancer predisposition genes, but highlight the major challenge of definitively validating candidates where the incidence of sporadic disease is high, germline mutations are not fully penetrant, and individual predisposition genes may only account for a tiny proportion of breast cancer families.

## Author Summary

Currently, we know that a woman who inherits a fault in one of two genes, *BRCA1* or *BRCA2*, has a high risk of developing both breast and ovarian cancer. However, such faults account for only half of all families with a strong family history of breast cancer. In this study, we planned to identify new genes that may be associated with an increased risk of developing breast cancer by looking for faults in every gene in the blood DNA of multiple women with breast cancer from large families with a strong family history of the condition over multiple generations. We can then track which gene fault is present in all the women with breast cancer in that family and in other families, but is not found in the women who did not develop breast cancer or have no family history. Using this approach, we identified faults in two genes, Fanconi C and Bloom helicase, in six families. Faults in these genes appear to increase the risk of developing breast cancer. Both these genes work in a similar way as *BRCA1* and *BRCA2*, and this highlights the importance of these functions in preventing breast cancer. Further studies need to be done to confirm our results.

## Introduction

Around one in six women who develop breast cancer has a first degree relative with the condition [1]. In the mid 1990s, a classical linkage approach identified germline mutations in two genes, *BRCA1* and *BRCA2*, which are associated with a high risk of developing both breast and ovarian cancer [2], [3]. Although *BRCA1* and *BRCA2*-specific genetic testing is rapidly evolving in the clinical setting, mutations in these genes are successful at explaining only around half of the dominant multi-case breast cancer only families [4], and their contribution to the heritable risk of breast cancer has been estimated to be no more than around 20% of the total [5], [6]. Importantly, the identification and management of individuals with high-risk breast cancer predisposition gene mutations is now well accepted in clinical practice. Although evidence-based risk management is only possible in a relatively small group of families, as it is limited by the identification of an underlying genetic mutation, the benefits for those individuals are well established [7].

Through a candidate gene approach, mutations in other high and moderate penetrance cancer-susceptibility genes have been identified in a further small proportion of families but the underlying etiology of the increased susceptibility to breast cancer in the majority of multi-case breast cancer families remains unknown. Recent advances in massively parallel sequencing technology have provided an agnostic means by which to efficiently identify germline mutations in individuals with inherited cancer syndromes at the individual family or cancer-specific level [8], [9]. The aim of this study is to identify through a whole exome sequencing approach, the underlying familial predisposition to breast cancer in multiple multi-generational breast cancer families in whom no *BRCA1* or *BRCA2* mutation was identified (*BRCA1/2* negative families), and to assess the candidate genes identified by this means in a cohort of familial *BRCA1/2* negative breast and ovarian cancer patients.

## Results/Discussion

We performed intra-family exome sequence analysis of multiple affected relatives from 15 high-risk, trans-generational breast cancer families in whom full *BRCA1* and *BRCA2* mutation analysis had been performed and was uninformative in at least one breast cancer-affected family member (Table 1). Sequencing was performed on GAIIx or HiSeq instruments (Illumina). The average read depth achieved for target regions was 83.19 and at least 80% (average 89.12%) of the capture target regions were covered by 10 or more sequence reads for all samples (Table S1). Following data filtering, an average of 35 overtly deleterious and 284 non-synonymous mutations were identified per individual (Table S1).

**Table 1:** Characteristics of 15 high-risk breast cancer families selected for exome sequencing.

Family	Mutation	Index 1^a^	Index 2	Index 3	Degree of relatedness	No. BC affected	No. OC affected	Other cancer types^b^
1	*FANCC* p.Arg179*	BC (46)	BC (37)	-	1	3	1	Pancreas, unspecified haematological
2	*FANCC* p.Arg185*	BC (36)	BC (45&53; bilat.)	-	1	4	0	Melanoma
3	*BLM* p.Gln645*	BC (39)	BC (39)	-	1	5	0	-
4	*PTEN* p.Glu73*	BC (35)	BC/OC (44)	-	5	11	2	Endometrium, prostate, cervix, unspecified haematological, stomach, unknown primary
5	*BRCA2* p.Leu1908fs	BC (43; male)	BC (43)	BC (53)	1, 5	15	1	lymphoma (5), prostate (4), melanoma (3), colorectal (2), bladder, endocrine, thyroid
6	*BRCA2* p.Pro9fs	BC (36)	BC (30)	-	3	5	0	Pancreas
7		BC (44; bilat.)	BC (34&50; bilat.)	-	3	3	0	Prostate
8		BC (46)	BC (39)	-	2	8	0	Acute leukaemia
9		BC (41)	BC (41)	-	1	9	0	Colorectal (3), throat (2)
10		BC (35&49; bilat.)	BC (42)	-	3	5	0	-
11		BC (54)	BC (37&43; bilat.)	-	1	5	0	Colorectal (2), prostate
12		BC (51)	BC (42), Lung (59)	-	4	6	2	Brain (2), cervix, lung, melanoma, pancreas, prostate
13		BC (38)	BC (39&44; bilat.)	-	1	4	0	Melanoma
14		BC (34)	BC (39), thyroid (32)	BC (45)	1, 3	4	0	Prostate (2), thyroid
15		BC (41), CRC (58)	BC (54)	BC (43)	1, 4	6	0	Colorectal (4), bladder, lymphoma, prostate, multiple myeloma

a Breast cancer (BC), ovarian cancer (OC), bilat. (bilateral). Age of diagnosis shown in parentheses.

b Other cancer types observed in family branch with apparent breast cancer risk (multiple cases shown in parentheses).

**Figure 1:**
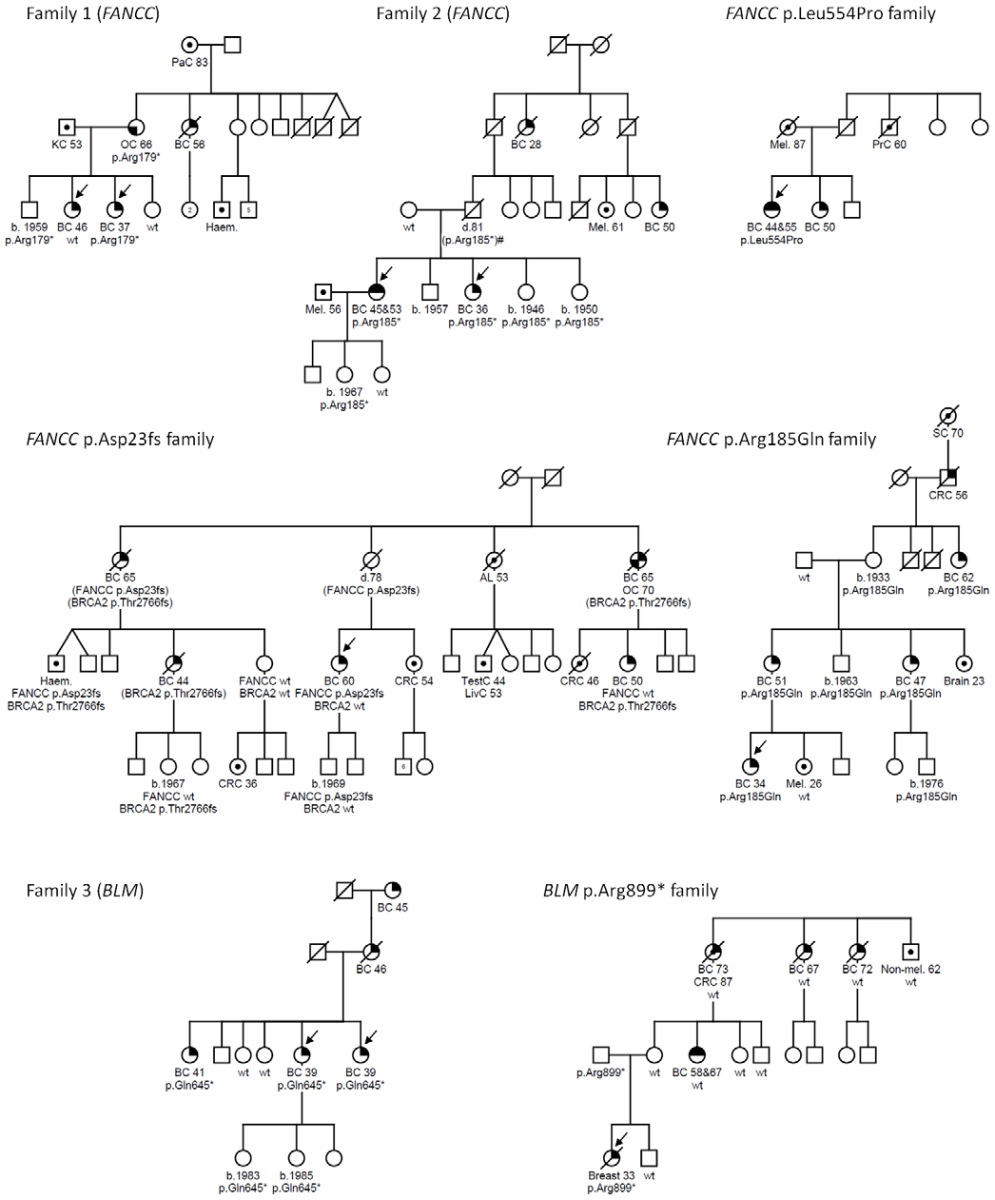
*FANCC* and *BLM* mutations identified in familial breast cancer pedigrees. Males and females are represented by squares and circles, respectively. Arrows indicate individuals who underwent whole exome sequencing (families 1–3) or were the index case in subsequent mutation analysis (*FANCC* p.Asp23fs, p.Leu554Pro and p.Arg185Gln and *BLM* p.Arg899* families). Cancer-affected individuals are represented with the following symbols: breast cancer, top right quadrant filled in; bilateral breast cancer, top half; ovarian cancer, bottom left quadrant; or other cancers as indicated, centre circle. Mutation status is indicated with either the family specific mutation or wildtype (wt) under each tested individual. Age at cancer diagnosis or year of birth (b.) where known is shown for all mutation carriers. Breast cancer (BC), ovarian cancer (OC), acute leukaemia (AL), colorectal cancer (CRC), haematological malignancy (type unspecified) (Haem.), kidney cancer (KC), liver cancer (LivC), melanoma (Mel.), pancreatic cancer (PaC), prostate cancer (PrC), skin non-melanoma (Non-mel.) stomach cancer (SC), testicular cancer (TestC). Mutations indicated in parentheses indicate untested obligate carriers. Family 2 contains an individual (indicated by #) for whom mutation status is inferred assuming that non-paternity or gonadal mosaicism have not occurred.

To identify candidate predisposition genes we only considered those with overtly deleterious mutations that were shared by multiple affected relatives and/or were targeted in more than one family and further priority was given to genes with a role in mechanistically well-established breast cancer–associated DNA repair. A list of all overtly deleterious mutations identified in among the 33 individuals sequenced is provided in Table S2. Two of the fifteen families were found to carry independent heterozygous truncating mutations in the Fanconi Anemia (FA) gene, *FANCC*. Neither family was reported to be of Ashkenazi Jewish ancestry and the mutations are different to those commonly reported among this ethnic group. Family 1 carried a novel nonsense mutation (*FANCC* c.535C>T, p.Arg179*) that was present in the youngest affected individual (breast cancer at age 37) and in her mother who had ovarian cancer at age 66, but not in her breast cancer-affected sister who was diagnosed at age 46 (Figure 1). Family 2 was found to harbor a known pathogenic FA mutation (*FANCC* c.553C>T, p.Arg185*) [10] which was present in two sisters who developed breast cancer aged 36, and bilateral breast cancer aged 46 and 53, respectively. A third family analyzed by exome sequencing was found to carry a heterozygous c.1993C>T mutation in the *BLM* gene which is predicted to truncate the protein at codon 645 (p.Gln645*). This known pathogenic Bloom syndrome mutation [11] co-segregated with cancer in the family (Figure 1), being present in all three sisters diagnosed with breast cancer aged 39, 39 and 41 years respectively and absent in the two unaffected sisters. Although retrospective likelihood segregation analysis of these limited pedigrees did not reach significance (see Text S1), overall, co-segregation of *FANCC* and *BLM* mutations in these families appears consistent with that expected for moderately penetrant breast cancer alleles.

Mutation analysis of all coding exons of *FANCC* and *BLM* was extended to the index cases from a further 438 *BRCA1/2* negative breast cancer families (from kConFab). This approach identified one further family with a heterozygous, known pathogenic *FANCC* mutation, (c.67delG, p.Asp23Ilefs*23, rs104886459) [12] and one with a heterozygous pathogenic *BLM* mutation (c.2695C>T, p.Arg899*) [11]. For *FANCC*, mutation hotspot exons 2, 5, 7, 14 and 15 were screened in the index cases from an additional 957 *BRCA1/2* uninformative breast cancer families attending familial cancer services (including 561 obtained from the Peter MacCallum Cancer Centre Familial Cancer Centre and a further 396 from kConFab). One further family with a heterozygous *FANCC* c.1661T>C (p.Leu554Pro, rs104886458) missense variant, which is a functionally validated pathogenic FA mutation, was identified [13].

The index case in the *FANCC* c.67delG family developed breast cancer at age 60 but independent clinical testing subsequently identified a deleterious mutation in *BRCA2* (c.8297delC, p.Thr2766Asnfs*11) in other breast cancer-affected family members (Figure 1). Genotyping of both mutations within this family suggests that different individuals may carry risk conferred by one or both of these family mutations.

The index case of the *FANCC* c.1661T>C family developed bilateral breast cancer at age 44 and 55, but DNA from other family members was not available for segregation analysis. All *FANCC* variants detected in index cases or controls are summarized in Table S3.

The index case of the *BLM* c.2695C>T family developed breast cancer at age 33 but segregation analysis showed the mutation was inherited from her father rather than her mother whose reported family history of breast cancer had initiated their recruitment into kConFab (Figure 1). Interestingly, breast cancer was diagnosed much earlier in the index case compared to her maternal relatives (33 years versus 58 to 73 years) possibly indicating a different genetic etiology. Unfortunately data regarding family history on the paternal side are limited. Neither the father nor the paternal grandparents were reported to have developed cancer but no further information regarding number or cancer status of other relatives is available. All *BLM* variants detected in index cases or controls are summarized in Table S4.

No pathogenic *BLM* mutations were detected in 464 healthy controls and none have been reported in the 1000 Genomes data (20100804 release, n = 1,092) [14] compared to 2/438 breast cancer families with *BLM* mutations. Likewise, no known pathogenic or overtly deleterious *FANCC* mutations were identified among the 464 controls or the 1000 Genomes data or among 654 healthy controls examined in an independent study [15]. The Exome Variant Server (EVS), NHLBI Exome Sequencing Project, Seattle, WA, does report deleterious mutations in *FANCC* and *BLM* in 3/3,510 and 4/3,510 individuals of European decent, respectively. However, this cohort includes extreme tail sampling of traits relating to heart, lung and blood disorders. The latter group in particular may be expected to show enrichment for mutations in DNA repair machinery including FA genes. Excluding the Exome Variant Server frequency data, a total of 4/1,395 breast cancer families screened for all or at least the mutation hot spot exons carried overtly deleterious *FANCC* mutations compared to none among the combined control population (n = 2,210). While this is indicative that overtly deleterious mutation in *FANCC* and *BLM* are likely to be very rare in the population this must be considered a crude measure as the controls were drawn from diverse populations the majority of which were not matched to the index cases. However, it is possible that more families in our breast cancer family cohort may be explained by *FANCC* and *BLM* mutations since, for both genes, private non-synonymous variants were identified that are predicted to be damaging by *in silico* algorithms. One such variant, for which there was DNA available for segregation analysis, was *FANCC* p.Arg185Gln. This variant closely segregated with disease in this family, which included four female blood relatives with breast cancers diagnosed at ages 34, 51, 47 and 62 (Figure 1). The p.Arg185Gln variant was identified in 1/1,395 breast cancer families but not in any of 464 controls and has not been reported in the 1000 Genomes project or EVS database.

Homozygous mutations in *FANCC* and *BLM* are responsible for FA (complementation group C) and Bloom syndrome, respectively, and individuals diagnosed with these syndromes have a high risk of cancer. Functionally, the FA and Bloom syndrome pathways play important roles in homologous recombination (HR) based repair of double-stranded DNA breaks [16], [17]. Constitutional inactivating mutations in genes integral to error-free HR and responsible for FA have been clearly associated with an increased susceptibility to both breast and ovarian cancer [16], and include the genes *BRCA1*, *BRCA2* (*FANCD1*), *FANCN* (*PALB2)*, *FANCJ* (*BRIP1*), *RAD51C* (*FANCO*) and *RAD51D*. Thus, in addition to the direct genetic evidence that we have described here, *FANCC* and *BLM* are strong candidates for breast cancer susceptibility genes due to their role in the precise regulation of HR and some of its associated functions. Although there is limited data, heterozygous *FANCC* mutations have previously been linked to an increased incidence of breast and early onset pancreatic cancer [15], [18], [19], however, no excess breast and ovarian cancer was observed among Ashkenazi Jews carrying the *FANCC* c.711+4A>T mutation [20]. While another previous study failed to identify overtly pathogenic *FANCC* mutations in breast cancer, the study cohort size was small (n = 88) [21]. In keeping with our data, two recurrent truncating mutations in the *BLM* gene were shown in a case control study to be associated with increased breast cancer risk in Russia [22]. Gruber *et al* reported an elevated risk of colorectal cancer in Ashkenazi Jews carrying the common *BLM* ^ASH^ mutation and a non-significant excess of breast cancer [23] although a later study failed to confirm these findings [24].

Further to the germline mutations in *FANCC* and *BLM*, exome sequencing identified mutations in the breast cancer predisposition genes, *PTEN* and *BRCA2* in an additional three of the original 15 families (Figure S1). The truncating *PTEN* mutation (c.217G>T, p.Glu73*) was identified in only one branch of the family suggesting another susceptibility gene may explain the extended family history. Prior to this finding, the treating familial cancer centre reported no *PTEN*-associated clinical features within the family. In family 5, exome sequencing identified a deleterious *BRCA2* mutation (c.5722_5723delCT, p.Leu1908Argfs*2, rs80359530) in two of the three family members tested (Figure S1). The mutation is present in a male diagnosed with breast cancer but not in the youngest affected female relative in the family, who had been offered the original clinical *BRCA1* and *BRCA2* mutation test in the clinic setting. Similarly in family 6, exome sequencing identified a deleterious *BRCA2* mutation (c.26delC, p.Pro9Glnfs*16, rs80359343) in a female diagnosed with breast cancer at age 30, but not in her cousin who was diagnosed at age 36 and was the only family member to have undergone full diagnostic *BRCA1* and *BRCA2* gene sequencing (Figure S1). These families are interesting in a clinical context since they were designated as unresolved on the basis of best clinical practice and demonstrate the need for targeted sequencing of all proven breast and ovarian cancer susceptibility genes to obtain maximum information in the clinical setting (as previously demonstrated [25]). Our data also highlights the major challenge confounding genetic studies of common adult onset familial disease; the presence of ‘phenocopies’ in families with an inherited genetic predisposition and/or the convergence of pedigrees with different genetic causes (*e.g. PTEN* family 4). Among the remaining nine breast cancer families there were numerous genes that were recurrently targeted that warrant further investigation. It is noteworthy that in one family, one individual harbored a known FA pathogenic truncating mutation in *FANCL*. Mutation of this gene is responsible for a very small fraction of FA families and only three pathogenic mutations in *FANCL* are recorded in the Fanconi Anemia Mutation Database.

In conclusion, we describe two potential breast cancer susceptibility genes *FANCC* and *BLM* both of which have functional roles in the regulation of HR. The heterozygous mutation carrier rate in Caucasians for these genes is extremely low (for *FANCC* it is estimated at 1/3,000 [15], whilst the carrier frequency of *BLM* mutations is unknown since the syndrome is exceedingly rare) and notwithstanding the possibility of the “winners curse” [26], the exome sequencing data is strongly suggestive that *FANCC* and *BLM* represent breast cancer predisposing genes. Together with the recently identified association of *RAD51* paralogues with cancer predisposition [27], [28], our findings suggest that the number of unidentified moderate to high-risk susceptibility genes is very much larger than previously expected and the number of families explained by each gene is likely to be much less than 1% (*cf. RAD51C* [27], [29]). Consequently, providing definitive evidence for a causative role for novel breast cancer genes will be challenging and will require validation of rare mutations in thousands rather than hundreds of families. We predict that this will be a generic problem associated with identifying causative mutations in common diseases such as breast cancer and that validation rather than the technical exercise of exome sequencing is where the real challenge lies.

## Materials and Methods

### Subjects

This study was approved by the Peter Mac Ethics Committee (project numbers 09/62 and 11/50). Informed consent was obtained from all participants. Fifteen high-risk breast cancer families with at least four cases of multi-generational breast cancer including at least one additional high-risk feature (such as bilateral, early onset or male breast cancer, or ovarian cancer) and at least two available blood specimens from breast cancer-affected individuals, were selected for whole exome sequencing from among approximately 800 *BRCA1* and *BRCA2* mutation negative families from the Kathleen Cunningham Foundation Consortium for Research into Familial Breast Cancer (kConFab), which has been collecting biospecimens and clinical and epidemiological information from families recruited through Familial Cancer Centres in Australia and New Zealand since 1997 [30]. DNA from two or three breast cancer-affected individuals were obtained from each family for analysis (as shown in Table 1), at least one of whom had previously been screened for *BRCA1* and *BRCA2* mutations (by sequencing of all coding exons and Multiplex Ligation-dependent Probe Amplification). Blood DNA from index cases from a further 834 mutation negative kConFab families and 561 mutation negative families obtained from the Peter MacCallum Cancer Centre Familial Cancer Centre were obtained for mutation analysis of candidate genes. Of those index cases obtained through the Familial Cancer Centre, individuals were breast cancer-affected, had a strong family history and been assessed for the probability of harboring a *BRCA1* or *BRCA2* mutations using BRCAPRO [31] and had been found on the basis of a verified family and personal history of having a 10% or greater probability. The index cases had undergone full diagnostic *BRCA1/2* mutation search and no mutation was identified. However, it should be noted that the majority of these families did not fulfill the very stringent family history criteria that was required for recruitment to kConFab, the research cohort from which the families for the initial exome sequencing were taken [30]. Non-cancer control DNA samples were obtained from kConFab (226 age- and ethnicity-matched best friend controls) and from the Princess Anne Hospital, UK (238 Caucasian female volunteers, as described previously [32]). DNA for candidate gene mutation analysis underwent whole genome amplification (WGA) using Repli-G Phi-mediated amplification system (Qiagen) prior to mutation analysis.

### Whole-Exome Sequencing

2–3 µg of DNA was fragmented to approximately 200 bp by sonication (Covaris) and used to prepare single- or paired-end libraries using the SPRIworks Fragment Library System I for Illumina Genome Analyzer on the SPRI-TE Nucleic Acid Extractor (Beckman Coulter). Exome enrichment was performed using the NimbleGen Sequence Capture 2.1 M Exome Array, EZ Exome Library (Roche NimbleGen) or SureSelect Human All Exon version 2 or 50 Mb libraries (Agilent Technologies) according to the recommended protocols. Sequencing was performed on GAIIx or HiSeq instruments (Illumina). Library preparation and sequencing details for each sample are provided in Table S1. We did not observe any significant differences in performance of the different exome capture platforms.

### Sequencing Alignment and Variant Calling

Paired-end sequence reads were aligned to the human genome (hg19 assembly) using the Burrows–Wheeler Aligner (BWA) program [33]. Local realignment around indels was performed using the Genome Analysis Tool Kit (GATK) software [34]. Subsequently, duplicate reads were removed using Picard and base quality score recalibration performed using GATK software. Single nucleotide variants (SNVs) and indels were identified using the GATK Unified Genotyper and variant quality score recalibration. Variants were annotated with information from Ensembl release 62 using Ensembl Perl Application Program Interface (API) including SNP Effect Predictor [35], [36]. Single-end sequence reads were aligned as above except duplicate reads were flagged prior to base quality score recalibration and included in variant calling.

### Candidate Variant Identification

Variants were first filtered for confident calls originating from bidirectional sequence reads using a quality threshold of ≥30, read depth of ≥10 and allele frequency ≥0.15. Prior to further filtering, variants were assessed for overtly deleterious mutation in known breast cancer associated genes [25]. Then, all variants present in the dbSNP database v132, except those also reported in the public version of the Human Gene Mutation Database (HGMD) [37] were removed, as were all common variants detected in >10 out of 33 exomes. Next, variants with functionally deleterious consequences (nonsense SNVs, frameshift indels, essential splice variants and complex indels) were identified for evaluation [35]. Functionally deleterious variants were evaluated in each individual as well as pairwise between relatives.

### Variant Validation Using Sanger Sequencing

Primers flanking the *BRCA2*, *PTEN*, *FANCC* and *BLM* mutations identified by whole exome sequence analysis were used to amplify germline DNA from affected index cases and all available relatives. The purified products were directly sequenced using BigDye terminator v3.1 chemistry on a 3130 Genetic Analyzer (Applied Biosystems).

### Mutation Analysis of *FANCC and BLM*

High resolution melt (HRM) analysis was performed on duplicate PCR products amplified from 15 ng WGA DNA. Primer sequences and PCR conditions are provided in Table S5. Melt analyses were performed on a LightCycler 480 Instrument using Gene Scanning Software (Roche). Duplicate PCR products exhibiting variant DNA melt curves were Sanger sequenced to identify sequence variations. All novel sequence variants were confirmed by Sanger sequencing an independent PCR amplified from non-WGA DNA. The functional effect of missense variants were evaluated using *in silico* prediction tools SIFT and PolyPhen-2 [38], [39].

### Accession Codes

The following GenBank reference sequences were used for variant annotation: *FANCC*, NM_000136 *BLM*, NM_000057; *PTEN*, NM_000314 and *BRCA2*, NM_000059.

### Web Resources

1000 Genomes Browser, http://browser.1000genomes.org/; Ensembl, http://www.ensembl.org/index.html; The Genome Analysis Toolkit, http://www.broadinstitute.org/gsa/wiki/index.php/The_Genome_Analysis_Toolkit; HGMD, http://www.hgmd.org/; Picard, http://picard.sourceforge.net; HGVS nomenclature for the description of sequence variants, http://www.hgvs.org/mutnomen/; NCBI SNP database, http://www.ncbi.nlm.nih.gov/projects/SNP/; The Fanconi Anemia Mutation Database, http://www.rockefeller.edu/fanconi/; BLMbase mutation registry, http://bioinf.uta.fi/BLMbase/; SIFT, http://sift.jcvi.org/; PolyPhen-2, http://genetics.bwh.harvard.edu/pph2/. Exome Variant Server, http://evs.gs.washington.edu/EVS/.

## Supporting Information

Figure S1 *PTEN and BRCA2* mutations identified in familial breast cancer pedigrees. Males and females are represented by squares and circles, respectively. The arrows indicate individuals who underwent whole exome sequencing (families 4–6). Cancer-affected individuals are represented with the following symbols: breast cancer, top right quadrant filled in; bilateral breast cancer, top half; ovarian cancer, bottom left quadrant; or other cancers as indicated, centre circle. Mutation status is indicated with either the family specific mutation or wildtype (wt) under each tested individual. Age at cancer diagnosis or year of birth (b.) where known is shown for all mutation carriers. Breast cancer (BC), ovarian cancer (OC), cervical cancer (CervC), colorectal cancer (CRC), cancer of unknown primary (CUP), endocrine cancer (EndoC), endometrial cancer (EndomC), haematological malignancy (type unspecified) (Haem.), lymphoma (Lymph), melanoma (Mel.), pancreatic cancer (PaC), prostate cancer (PrC), stomach cancer (SC), thyroid cancer (ThyrC). Mutations indicated in parentheses indicate untested obligate carriers.
(PDF)

Table S1 Whole exome sequencing performance and variant count. ^a^Indicates adaptor type (and protocol) used for library preparation. Paired-end (PE), single-end (SE), multiplex paired-end (in PE). ^b^Libraries were prepared by hand (manual) or using the SPRIworks Fragment Library System (SPRIworks) incorporating size selection as indicated in parentheses. ^c^Exome enrichment was performed using either the NimbleGen Sequence Capture 2.1 M Exome Array (NG exome array), EZ Exome Library version 1 or 2 (NG exome array v1 or v2) or Agilent SureSelect Human All Exon version 2 (Ag Exome v2) or 50 Mb (Ag Exome 50 Mb) libraries. ^d^Percentage of reads that map and align to the reference genome and overlap with the targeted bases by at least one base. The target regions differ according to the capture method used. ^e^Number of variants shared by all exome-sequenced family members (i.e. either 2 or 3).
(XLSX)

Table S2 Overtly deleterious variants identified by exome sequencing. ^a^Genomic position (chromosome_nucleotide) of the reference nucleotide for each variant provided relative to human geneome reference assembly GRCh37 (hg19). ^b^Reference nucleotide sequence. ^c^Alternate (or variant) nucleotide sequence detected by exome sequencing. This variant list has not been extensively validated by Sanger sequencing and may include sequencing artefacts. ^d^Predicted consequence of variant relative to ensembl transcript provided. Only variants with an overtly deleterious predicted consequence (i.e. stop_gained, frameshift_coding, complex_indel or essential_splice_site) are included in this list. ^e^Number of times variant detected among exome data from 33 individuals. Only those variants present in fewer than 10/33 individuals are included in this list. ^f^Number of families in whom variant was detected. ^g^Number of times variant was detected in an individual who also carried a *FANCC* or *BLM* mutation. ^h^Predicted position of alteration relative to protein associated with ensembl transcript. ^i^Predicted amino acid change (provided for SNVs only). ^j^dbSNP IDs for previously identified variants co-ocurring at the same variant position (not matched for nucleotide change). Variants with dbSNP references were filtered from this list with the exception of variants in known breast cancer genes (*i.e. BRCA1, BRCA2, ATM, CHEK2, BRIP, PALB2 etc.*) or variants reported in the HGMD public database. ^k^Other existing variation IDs, primarily from the HGMD public database.
(XLSX)

Table S3 Variants identified by HRM-based mutation analysis of *FANCC.* ^a^Exon (Ex), intervening sequence (IVS). ^b^Variant positions are reported in reference to GenBank reference sequence NM_000136.2 (mRNA). ^c^In addition to the non-coding variants listed, dbSNP rs4647534 (c.1155-38T>C) were detected at high frequency in both cases and controls. ^d^All variants were queried against 1000 Genomes (1000 G) data using the 1000 Genomes Browser (http://browser.1000genomes.org/index.html), which integrates SNP and indel calls from 1,092 individuals (data release 20110521 v3). The minor allele frequency (MAF) is provided here. ^e^All variants were queried against Exome Variant Server (EVS), NHLBI Exome Sequencing Project (ESP) (http://evs.gs.washington.edu/EVS/) [May 2012]. EVS contains SNP information from 5,379 individuals (data release ESP5400). The minor allele frequency (MAF) is provided here. ^f^All variants were queried against the public version of the Human Gene Mutation Database (HGMD, http://www.hgmd.cf.ac.uk/). ^g^All variants were queried against the Fanconi Anemia Mutation Database (http://www.rockefeller.edu/fanconi/). The number in parentheses indicates the number of times this variant has been reported in FA cases. ^h^The numbers of samples examined varied by exon, as indicated for each variant.
(XLSX)

Table S4 Variants identified by HRM-based mutation analysis of *BLM.* ^a^Exon (Ex), intervening sequence (IVS). ^b^Variant positions are reported in reference to GenBank reference sequence NM_000057.2 (mRNA). ^c^In addition to the variants listed, dbSNPs rs11852361 (c.2603C>T), rs7167216 (c.3961G>A), rs2227933 (c.3102G>A), rs2227934 (c.3531C>A), rs1063147 (c.3945C>T), rs28385029 (c.2193+61G>C), rs17181698 (c.2193+84C>T), rs17273206 (c.2308-50G>A), rs3815003 (c.2555+7T>C), rs17273842 (c.3358+32T>G) were detected at high frequency in cases or 1000 Genomes data. ^d^All variants were queried against 1000 Genomes (1000 G) data using the 1000 Genomes Browser (http://browser.1000genomes.org/index.html), which integrates SNP and indel calls from 1,092 individuals (data release 20110521 v3). The minor allele frequency (MAF) is provided here. ^e^All variants were queried against Exome Variant Server (EVS), NHLBI Exome Sequencing Project (ESP) (http://evs.gs.washington.edu/EVS/) [May 2012]. EVS contains SNP information from 5,379 individuals (data release ESP5400). The minor allele frequency (MAF) is provided here. ^f^All variants were queried against the public version of the Human Gene Mutation Database (HGMD, http://www.hgmd.cf.ac.uk/). ^g^All variants were queried against the BLMbase Mutation Registry (http://bioinf.uta.fi/BLMbase/). The number in parentheses indicates the number of times this variant has been reported in FA cases.
(XLSX)

Table S5 Primers used for mutation analyses of *FANCC* and *BLM.* The list of forward and reverse primers used for mutation analyses of *FANCC* and *BLM.*
(XLSX)

Text S1 Retrospective likelihood segregation analysis methods and data.
(DOCX)
