# Supplementary figures and images for: Exome Sequencing Identifies Rare Deleterious Mutations in DNA Repair Genes FANCC and BLM as Potential Breast Cancer Susceptibility Alleles
Source: PLoS Genet. 2012 Sep 27;8(9):e1002894. doi: 10.1371/journal.pgen.1002894 (PMC3459953; doi:10.1371/journal.pgen.1002894)

Family 4 (*PTEN*)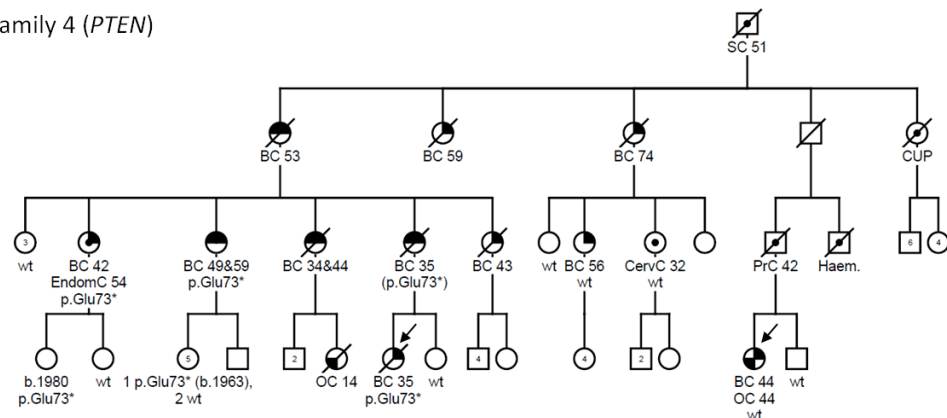Family 6 (*BRCA2*)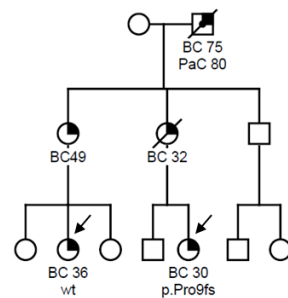Family 5 (*BRCA2*)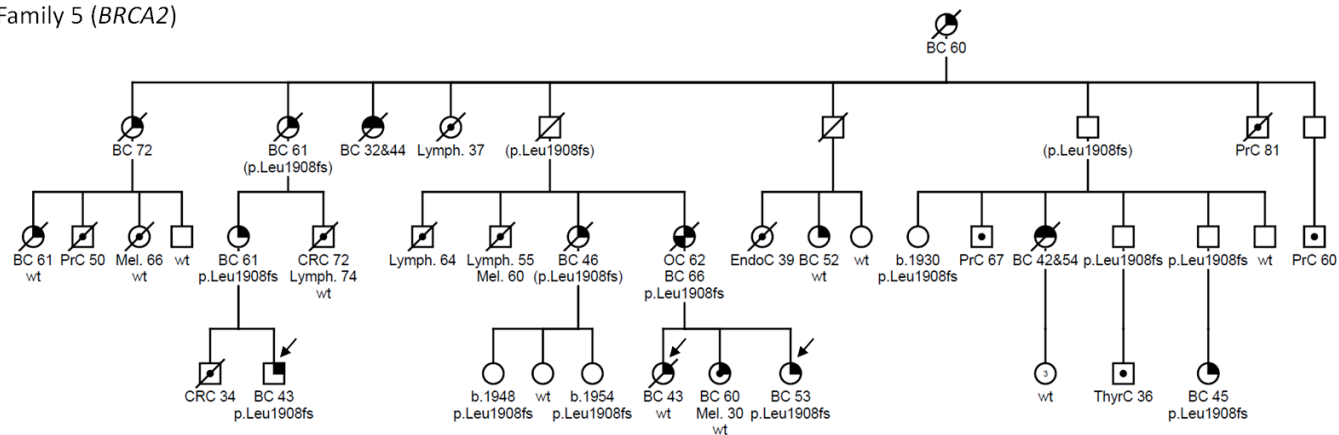

Supplement: Figure S1 — PTEN and BRCA2 mutations identified in familial breast cancer pedigrees. Males and females are represented by squares and circles, respectively. The arrows indicate individuals who underwent whole exome sequencing (families 4–6). Cancer-affected individuals are represented with the following symbols: breast cancer, top right quadrant filled in; bilateral breast cancer, top half; ovarian cancer, bottom left quadrant; or other cancers as indicated, centre circle. Mutation status is indicated with either the family specific mutation or wildtype (wt) under each tested individual. Age at cancer diagnosis or year of birth (b.) where known is shown for all mutation carriers. Breast cancer (BC), ovarian cancer (OC), cervical cancer (CervC), colorectal cancer (CRC), cancer of unknown primary (CUP), endocrine cancer (EndoC), endometrial cancer (EndomC), haematological malignancy (type unspecified) (Haem.), lymphoma (Lymph), melanoma (Mel.), pancreatic cancer (PaC), prostate cancer (PrC), stomach cancer (SC), thyroid cancer (ThyrC). Mutations indicated in parentheses indicate untested obligate carriers. (PDF) [file pgen.1002894.s001.pdf]
