# Supplementary material for: Exome Sequencing Identifies Rare Deleterious Mutations in DNA Repair Genes FANCC and BLM as Potential Breast Cancer Susceptibility Alleles
Source: PLoS Genet. 2012 Sep 27;8(9):e1002894. doi: 10.1371/journal.pgen.1002894 (PMC3459953; doi:10.1371/journal.pgen.1002894)
Supplement: Text S1 — Retrospective likelihood segregation analysis methods and data. (DOCX) [file pgen.1002894.s007.docx]

**Text S1. Retrospective likelihood segregation analysis.**

Two individuals from one family tested positive for a *BRCA2* mutation and, therefore, the family was excluded from analysis. Analyses for *FANCC* were performed on three families (families 1 and 2, and the p.Arg185Gln family), and analyses for *BLM* were performed on two families (family 3 and the p.Arg899* family). The censoring process adopted was such that individuals were censored as affected at age at breast cancer diagnosis or if they had undergone a bilateral mastectomy as a treatment measure (i.e. within a year prior to breast cancer diagnosis). Otherwise individuals were censored as unaffected at age of mastectomy if it occurred >1 year before breast cancer diagnosis, age at other cancer diagnosis, age at last follow-up or age at death, whichever occurred first. Individuals with censoring ages >80-years were censored as unaffected at age 80-years.

A retrospective likelihood segregation analysis was employed to account for non-random ascertainment of families. We assumed disease incidence followed a proportional hazards model. We modelled the likelihood of observing the family’s genotypes and phenotypes conditional on observing the genotypes of the first tested individual(s) from a family and all family phenotypes.

| **Gene** | **Families** | **Individuals** | **log-HR** | **se** | **HR (95% CI)** |
| --- | --- | --- | --- | --- | --- |
| *FANCC* | 3 | 163 | 1.649 | 1.001 | 5.20 (0.73-37.00) |
| *BLM** | 2 | 127 | - | - | - |

HR = hazard ratio; se = standard error; CI = confidence interval

* Unable to produce estimates – the likelihood did not maximise
